# The DNA-dependent protein kinase catalytic subunit promotes sepsis-induced cardiac dysfunction through disrupting INF-2-dependent mitochondrial dynamics

**DOI:** 10.7150/ijms.91894

**Published:** 2024-02-12

**Authors:** Mudi Ma, Hao Zhou, Ying Zhang, Woliang Yuan, Chaoxiong Chen

**Affiliations:** 1 Shenshan Medical Center, Sun Yat-sen Memorial Hospital, Sun Yat-sen University, Shanwei, Guangdong, China.; 2 Sun Yat-sen Memorial Hospital, Sun Yat-sen University, Guangzhou, China.; 3 Senior Department of Cardiology, The Sixth Medical Center of People's Liberation Army General Hospital, Beijing, China.

**Keywords:** DNA-PKcs, INF2, LPS, mitochondrial homeostasis

## Abstract

Sepsis-induced cardiomyopathy (SIC) represents a severe complication of systemic infection, characterized by significant cardiac dysfunction. This study examines the role of DNA-dependent protein kinase catalytic subunit (DNA-PKcs) and Inverted Formin 2 (INF2) in the pathogenesis of SIC, focusing on their impact on mitochondrial homeostasis and dynamics. Our research demonstrates that silencing DNA-PKcs alleviates lipopolysaccharide (LPS)-induced cardiomyocyte death and dysfunction. Using HL-1 cardiomyocytes treated with LPS, we observed that DNA-PKcs knockdown notably reverses LPS-induced cytotoxicity, indicating a protective role against cellular damage. This effect is further substantiated by the reduction in caspase-3 and caspase-9 activation, key markers of apoptosis, upon DNA-PKcs knockdown. Besides, our data further reveal that DNA-PKcs knockdown attenuates LPS-induced mitochondrial dysfunction, evidenced by improved ATP production, enhanced activities of mitochondrial respiratory complexes, and preserved mitochondrial membrane potential. Moreover, DNA-PKcs deletion counteracts LPS-induced shifts towards mitochondrial fission, indicating its regulatory influence on mitochondrial dynamics. Conclusively, our research elucidates the intricate interplay between DNA-PKcs and INF2 in the modulation of mitochondrial function and dynamics during sepsis-induced cardiomyopathy. These findings offer new insights into the molecular mechanisms underpinning SIC and suggest potential therapeutic targets for mitigating mitochondrial dysfunction in this critical condition.

## Introduction

Sepsis, a life-threatening organ dysfunction caused by a dysregulated host response to infection, often leads to sepsis-induced cardiomyopathy (SIC), a condition marked by myocardial depression and ventricular dilatation 1, 2. Mitochondria, the cellular powerhouses, play a crucial role in cardiac energy metabolism and are increasingly recognized as central players in the pathogenesis of SIC 3, 4. Mitochondria, quintessential to cellular metabolism and energy production, are increasingly recognized as pivotal in the pathogenesis of sepsis-induced cardiomyopathy (SIC) 5, 6. In this condition, systemic inflammatory response to infection precipitates profound myocardial dysfunction. Central to this dysfunction is mitochondrial impairment, manifesting through disrupted oxidative phosphorylation, leading to a critical reduction in ATP production, essential for myocardial energetics 7, 8. Concurrently, exacerbated generation of mitochondrial reactive oxygen species (ROS) contributes to oxidative stress, further exacerbating myocardial injury 9. Moreover, the opening of the mitochondrial permeability transition pore (mPTP) under septic conditions, often due to calcium overload and oxidative stress, triggers cardiomyocyte apoptosis and necrosis 10. Intriguingly, mitochondrial dynamics, including fission and fusion processes, are markedly altered in SIC, impacting mitochondrial quality control 11, 12. Dysregulated mitophagy, the selective autophagic clearance of damaged mitochondria, becomes apparent, leading to the accumulation of dysfunctional mitochondria and aggravating cardiac dysfunction 12. Understanding these mitochondrial mechanisms in SIC not only sheds light on the complex pathophysiology of sepsis but also opens avenues for novel therapeutic interventions targeting mitochondrial pathways. This could potentially mitigate cardiac dysfunction in sepsis, a major challenge in critical care.

DNA-dependent protein kinase catalytic subunit (DNA-PKcs), a key player in DNA repair, has emerged as a significant factor in inflammatory diseases. Beyond its canonical role in the non-homologous end joining (NHEJ) pathway, DNA-PKcs influences inflammatory signaling pathways. Recent studies reveal that DNA-PKcs activation can exacerbate inflammatory responses, potentially by modulating the activity of key transcription factors like NF-κB 13. This modulation contributes to the upregulation of pro-inflammatory cytokines, driving the pathology of various inflammatory conditions 13. Conversely, DNA-PKcs inhibition has shown promise in attenuating these inflammatory responses, highlighting its therapeutic potential 14. The intricate balance between DNA-PKcs's roles in maintaining genomic integrity and promoting inflammation underscores its dual significance in both cellular survival and disease pathogenesis. This duality presents a complex yet intriguing target for therapeutic intervention in inflammatory diseases. Emerging research underscores DNA-PKcs's pivotal role in mediating cellular responses within cardiac and vascular tissues. In the context of heart disease, DNA-PKcs is implicated in the regulation of cardiomyocyte survival and apoptosis, particularly under stress conditions such as ischemia 15. Its activation is linked to exacerbated tissue damage following myocardial infarction, suggesting a potential target for therapeutic intervention 15. In vascular diseases, DNA-PKcs contributes to endothelial dysfunction and atherosclerosis 16. It influences the balance between endothelial repair and injury, and modulates inflammatory responses in atherosclerotic plaques 17. This dual role of DNA-PKcs in both myocardial and vascular pathology marks it as a critical factor in the broader spectrum of cardiovascular diseases, offering a novel avenue for targeted therapies.

Inverted Formin 2 (INF2), a member of the formin family of proteins, has recently been identified as a critical regulator in mitochondrial and cardiac function. INF2's unique role in modulating mitochondrial shape and dynamics is pivotal for cardiac health 18, 19. Dysregulation of INF2 has been linked to mitochondrial dysfunction, characterized by altered mitochondrial fission and fusion balance, essential for maintaining cellular energy homeostasis 20. In cardiac tissues, this dysregulation contributes to impaired mitochondrial function, a key factor in the pathogenesis of various heart diseases, including cardiomyopathy and heart failure 21, 22. Recent studies suggest that INF2-mediated mitochondrial abnormalities can lead to kidney fibrosis 23. Understanding INF2's role in mitochondrial dynamics provides valuable insights into the molecular mechanisms underlying cardiac pathologies and opens new therapeutic avenues for treating heart diseases, emphasizing its potential as a novel target for cardiac therapeutics. The aim of our study is to figure out whether DNA-PKcs affected the progression of septic cardiomyopathy through controlling mitochondrial homeostasis with a focus on INF2.

## Methods

### Cell culture and transfection

HL-1 cells were cultured in DMEM medium (ScienCell, San Diego, CA, USA) supplemented with 5% fetal bovine serum (FBS), 100 U/mL penicillin, and 100 mg/mL streptomycin at 37 °C in a 5% CO_2_ humidified atmosphere. Experiments were conducted using cells between passages 2-5. Lentiviruses (Genechem, Shanghai, China) were used to knockdown DNA-PKcs and INF2 in HL-1 cells. Lentiviruses expressing DNA-PKcs shRNA (Lenti-shDNA-PKcs), INF2 shRNA (Lenti-shINF2), or control (Lenti-Ctrl) were infected into the cells at a multiplicity of infection (MOI) of 10, following the standard procedure. Cells were exposed to 10 μg/ml LPS for 24 hours to induce an in vitro septic cardiomyopathy model.

### Quantitative reverse transcription-polymerase chain reaction (qRT-PCR)

For quantitative reverse transcription-polymerase chain reaction (qRT-PCR), total RNA was extracted using the TRIzol reagent (Invitrogen, Carlsbad, CA, USA) and reverse transcribed into cDNA using the TaKaRa Biotechnology reverse transcription kit (Dalian, China). The SYBR premix ExTaqII (TaKaRa Biotechnology, Dalian, China) and Applied Biosystems 7500 Real-time PCR system (Foster City, California, USA) were utilized for qPCR analysis. β-actin served as the reference control during the analysis.

### Western blotting

For Western blotting analysis, total protein extraction was carried out using the radio immunoprecipitation assay (RIPA) buffer (Beyotime, Shanghai, China) supplemented with 10 mM phenylmethylsulfonyl fluoride (PMSF, Sigma-Aldrich). The BCA protein assay kit (Thermo Fisher Scientific, Rockford, Illinois, USA) was used to quantify the proteins. Subsequently, 5 × loading buffer (Beyotime) was added to the samples, which were then boiled for 10 minutes. The samples were separated by sodium dodecyl sulfate-polyacrylamide gel electrophoresis (SDS-PAGE) and transferred onto polyvinylidene fluoride (PVDF) membranes. Following this, the membranes were incubated with primary antibodies, washed, and then incubated with horseradish peroxidase (HRP)-conjugated secondary antibodies. Protein bands were visualized using an enhanced chemiluminescence (ECL) system (Clinx Science Instruments, Shanghai, China) and quantified using ImageJ software (Rawak Software, Stuttgart, Germany).

### Mitochondrial membrane potential and mPTP opening rate measurement

For the assessment of mitochondrial membrane potential, the mitochondrial membrane potential assay kit with JC-1 (C2006, Beyotime, China) was utilized following the manufacturer's instructions. HL-1 cells were incubated with fresh medium and an equal volume of JC-1 staining solution at 37 °C for 20 minutes. After washing, the fluorescence intensity was measured using fluorescence microscopy (FV1000, Olympus). The opening rate of mPTP was determined as previously described 24.

### ELISA

ELISA was utilized to measure the activities of caspase-3, caspase-9, mitochondrial respiratory complex I, mitochondrial respiratory complex II, mitochondrial respiratory complex III, and ATP production in HL-1 cells. The ELISA Kits were employed following the manufacturer's instructions. Briefly, 50 μL samples were mixed with 50 μL of the reaction mixture and incubated at 37°C for 40 minutes. The OD450nm values at 10 minutes and 40 minutes were recorded using a multi-plate reader. Additionally, the OD450nm value at the endpoint of the standard was recorded for the preparation of the standard curve. The results were calculated based on the standard curve, reaction time, and sample volume.

### Statistics

GraphPad Prism 8 was utilized for all statistical analysis and graph generation. To assess dataset normality, the Shapiro-Wilk test was employed for all statistical tests conducted. The specific statistical test used for each dataset is indicated in the corresponding figure legend. In cases where the data did not pass the normality test, the corresponding non-parametric test was utilized. Statistical significance was defined as p<0.05. The number of biological replicates (i.e., number of animals) is denoted as N, while the number of technical replicates (i.e., the number of cells) analyzed is represented by n. For datasets that meet the assumption for hierarchical analysis, "nested" tests were performed. Unless explicitly mentioned as a nested analysis, all statistical tests treated each analyzed cell as an individual data point. Dot plots representing different animals or biological replicates are distinguished by different shapes.

## Results

### DNA-PKcs silencing alleviates LPS-mediated cardiomyocyte death and dysfunction

**Figure 1:**
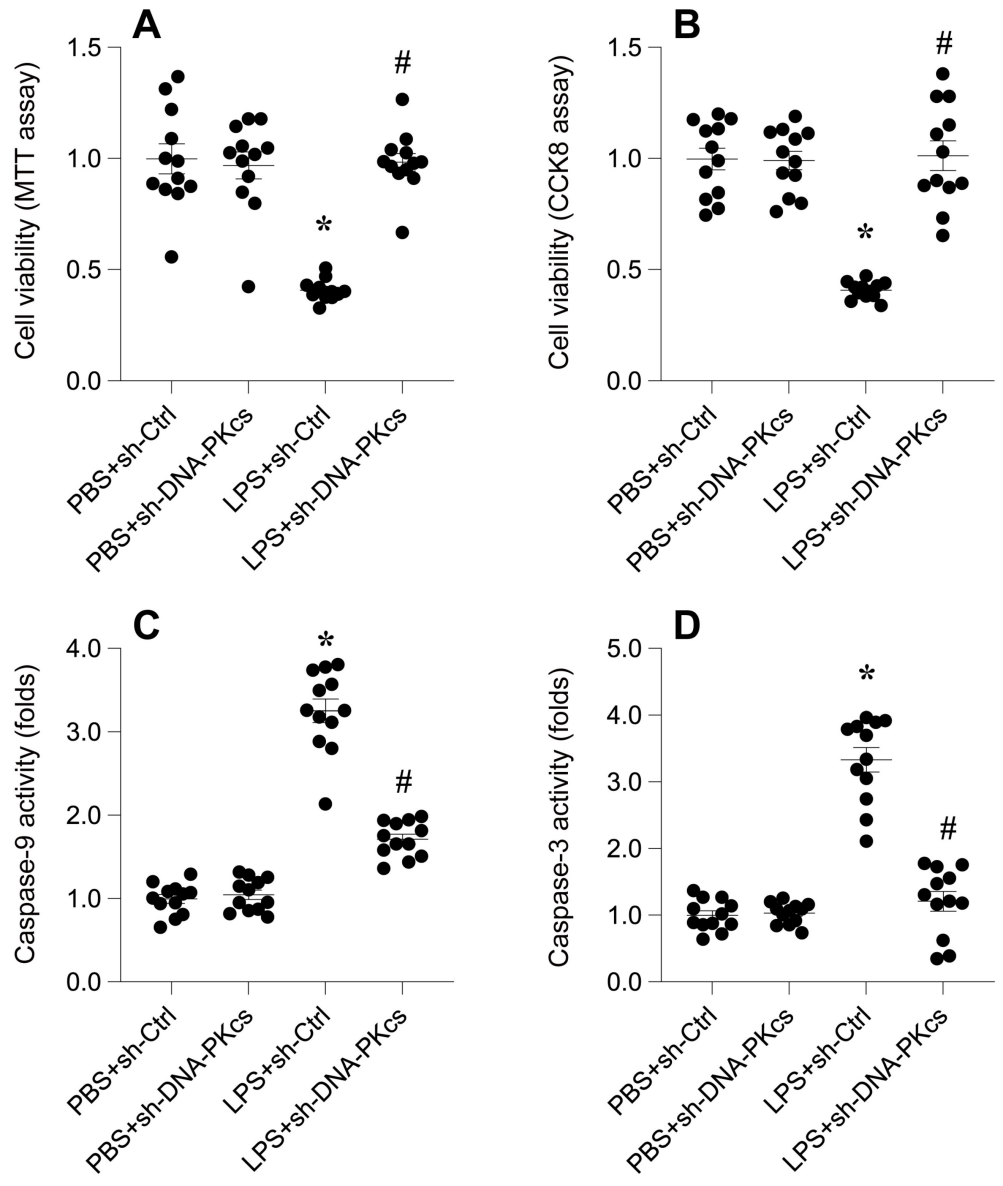
** DNA-PKcs knockdown alleviates LPS-mediated cardiomyocyte death and dysfunction. A.** MTT assay was used to evaluate the cell viability of HL-1 cells in the presence of LPS. shRNA against DNA-PKcs was transfected into HL-1 cells before LPS treatment. **B.** CCK-8 assay was applied to analyze the cell viability of HL-1 cells upon LPS exposure. shRNA against DNA-PKcs was transfected into HL-1 cells before LPS treatment. **C-D.** ELISA kits were used to analyze the activities of caspase-3 and caspase-9 in the presence of LPS. *p<0.05 vs. PBS+sh-Ctrl group, #p<0.05 vs. LPS+sh-Ctrl group.

To elucidate the impact of DNA-PKcs on lipopolysaccharide (LPS)-induced septic cardiomyopathy, we employed an in vitro model using HL-1 cardiomyocytes. Cells were exposed to 10 μg/ml LPS for 24 hours, followed by assessment of cell viability using MTT and CCK-8 assays. Results depicted in Figure 1A-B reveal a significant reduction in cell viability post-LPS treatment compared to phosphate-buffered saline (PBS)-treated controls, indicating a detrimental effect of LPS on cardiomyocyte survival.

To determine the involvement of DNA-PKcs in this LPS-induced viability decrement, HL-1 cells were transfected with shRNA targeting DNA-PKcs prior to LPS exposure. Subsequent viability assays demonstrated a notable reversal of LPS-induced cytotoxicity upon DNA-PKcs knockdown (Figure 1A-B), suggesting a protective role against LPS-mediated cellular damage.

Further investigation into the apoptotic pathways was conducted through ELISA-based quantification of caspase-3 and caspase-9 activities. Figures 1C-D show a marked activation of these caspases in LPS-treated cells, indicative of apoptosis induction. Intriguingly, DNA-PKcs knockdown significantly abrogated this activation, underscoring its role in modulating apoptosis in septic cardiomyopathy (Figure 1C-D).

Collectively, these findings underscore DNA-PKcs as a crucial mediator in LPS-induced cardiomyocyte dysfunction, offering new insights into the molecular dynamics of septic cardiomyopathy and potential therapeutic targets.

### DNA-PKcs knockdown attenuates LPS-caused mitochondrial dysfunction

In this study, we investigated whether DNA-PKcs is implicated in LPS-mediated cardiomyocyte dysfunction via its impact on mitochondrial homeostasis. Mitochondrial function was analyzed following LPS exposure in HL-1 cardiomyocytes, with a particular focus on ATP production, mitochondrial respiratory complex activities, membrane potential, and apoptotic markers.

**Figure 2:**
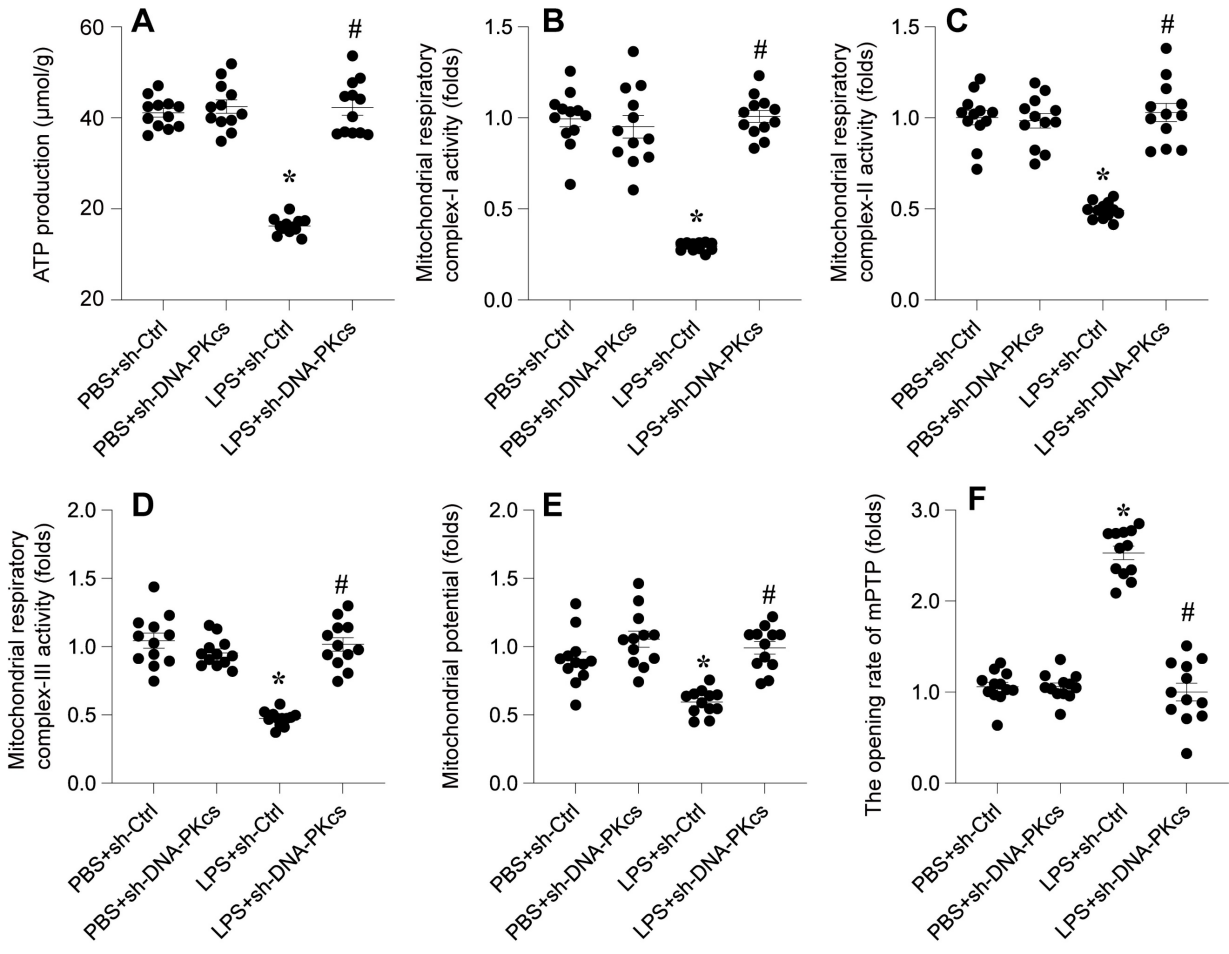
** DNA-PKcs knockdown attenuates LPS-caused mitochondrial dysfunction. A.** ATP production was determined by ELISA. **B-D.** The activities of mitochondrial respiratory complex I, II, III were measured by ELISA. **E.** Mitochondrial potential was evaluated through immunofluorescence assay and the relative changes of mitochondrial potential was evaluated. **F.** The mPTP opening rate was determined by an ELIST kit. *p<0.05 vs. PBS+sh-Ctrl group, #p<0.05 vs. LPS+sh-Ctrl group.

Initially, a rapid decrease in ATP production was observed upon LPS treatment, a change that was significantly reversed upon the knockdown of DNA-PKcs (Figure 2A). Given the crucial role of mitochondrial respiration in ATP synthesis, we employed ELISA kits to assess the activities of mitochondrial respiratory complexes I, II, and III. Post-LPS exposure, these complexes exhibited marked suppression in their activities (Figure 2B-D). However, in HL-1 cells transfected with shRNA targeting DNA-PKcs, this repression was notably mitigated, indicating a protective role of DNA-PKcs knockdown against LPS-induced mitochondrial dysfunction.

Moreover, mitochondrial membrane potential, a critical indicator of mitochondrial health, was also compromised following LPS exposure (Figure 2E). Intriguingly, this alteration was not observed in cells with DNA-PKcs knockdown. Additionally, we examined mitochondrial apoptosis through the opening dynamics of the mitochondrial permeability transition pore (mPTP). LPS treatment significantly increased the opening rate of mPTP, a response that was attenuated by the targeted knockdown of DNA-PKcs (Figure 2F).

Collectively, these findings reveal a crucial association between LPS-mediated mitochondrial dysfunction and DNA-PKcs activation. This connection underscores the potential role of DNA-PKcs in modulating mitochondrial integrity and function in the context of cardiomyocyte response to septic stimuli.

### DNA-PKcs knockdown attenuates LPS-caused mitochondrial dynamics disorder through affecting INF2 expression

**Figure 3:**
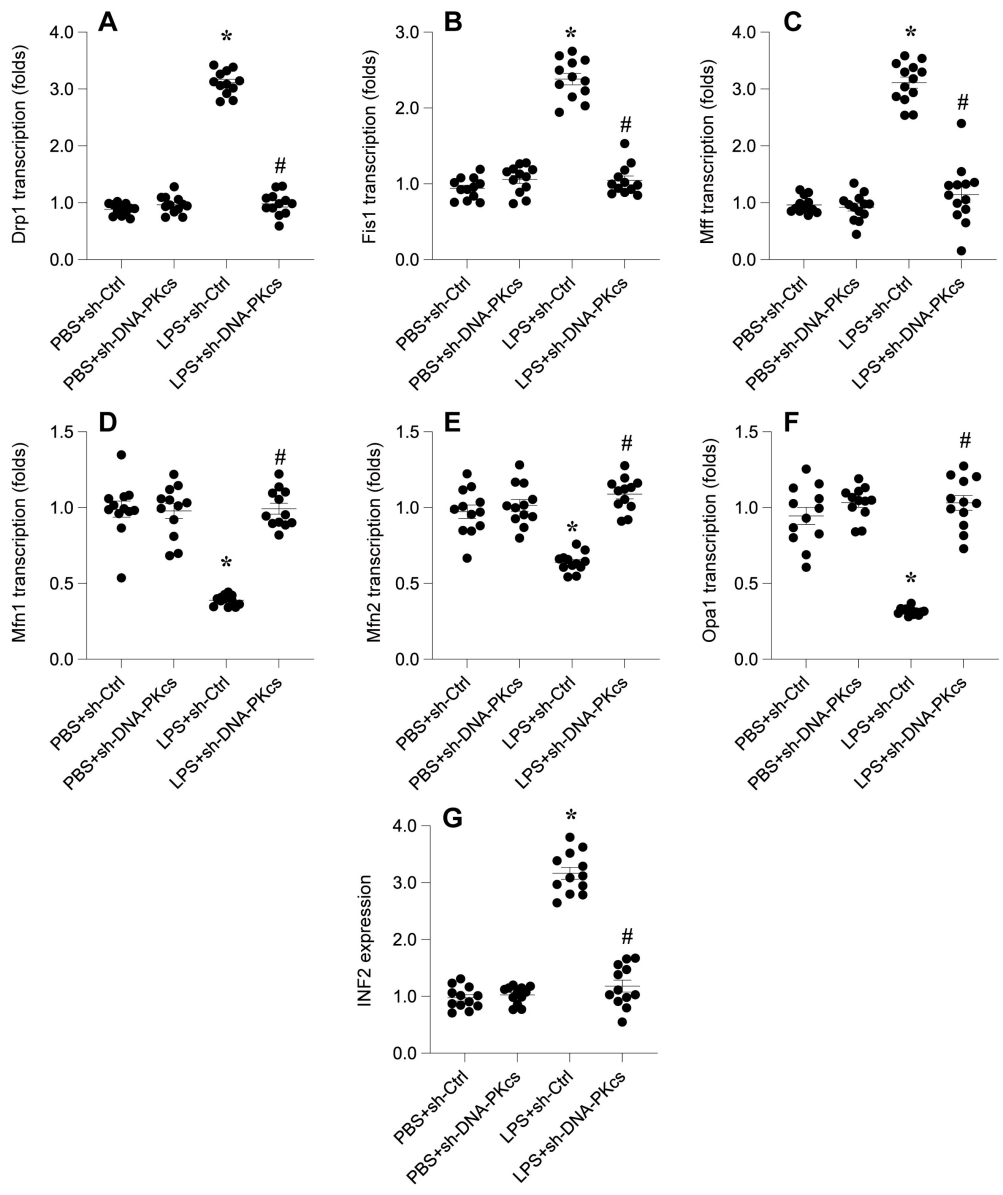
** DNA-PKcs knockdown attenuates LPS-caused mitochondrial dynamics disorder through affecting INF2 expression. A-F.** RNA was isolated from HL-1 cells and the transcriptions of Drp1, Fis1, Mff, Mfn1, Mfn2 and Opa1 were measured by qPCR. **G.** Proteins were collected from HL-1 cells and the expression of INF2 was measured by western blots. *p<0.05 vs. PBS+sh-Ctrl group, #p<0.05 vs. LPS+sh-Ctrl group.

In this investigation, we probed the relationship between DNA-PKcs-mediated mitochondrial dysfunction and abnormal mitochondrial dynamics, a critical factor in cellular homeostasis. Through quantitative PCR (qPCR) analysis, we observed that lipopolysaccharide (LPS) exposure significantly upregulated the transcription of mitochondrial fission genes Drp1, Fis1, and Mff. Concurrently, the mRNA expression of mitochondrial fusion genes Mfn1, Mfn2, and Opa1 was downregulated (Figure 3A-F), indicating an LPS-induced shift towards mitochondrial fission over fusion. These alterations suggest an imbalance in mitochondrial dynamics favoring fission processes in response to LPS.

Intriguingly, the deletion of DNA-PKcs appeared to counteract these effects, preventing the LPS-induced promotion of mitochondrial fission and facilitating the reversal of mitochondrial fusion (Figure 3A-F). This observation points to a regulatory role of DNA-PKcs in mitochondrial dynamics under septic conditions.

Further, we explored the potential involvement of Inverted Formin 2 (INF2) in DNA-PKcs-mediated mitochondrial dynamic disorders. Western blot analysis was employed to examine the expression of INF2 following LPS treatment and subsequent DNA-PKcs knockdown. As illustrated in Figure 3G, LPS treatment significantly elevated INF2 expression compared to the PBS control group, an effect that was notably disrupted by shRNA-mediated knockdown of DNA-PKcs.

Collectively, these findings elucidate the influence of DNA-PKcs on mitochondrial dynamics during LPS exposure, potentially mediated through the regulation of INF2. This sheds light on the complex interplay between DNA-PKcs and mitochondrial dynamics, offering new insights into the molecular mechanisms underpinning mitochondrial dysfunction in septic conditions.

### INF2 knockdown attenuates LPS-caused mitochondrial dynamics disorder

In this study, we sought to determine the involvement of Inverted Formin 2 (INF2) in lipopolysaccharide (LPS)-induced mitochondrial dynamics disorder. HL-1 cardiomyocytes were transfected with shRNA targeting INF2 to investigate its role in the regulation of mitochondrial fission and fusion processes under septic conditions.

**Figure 4:**
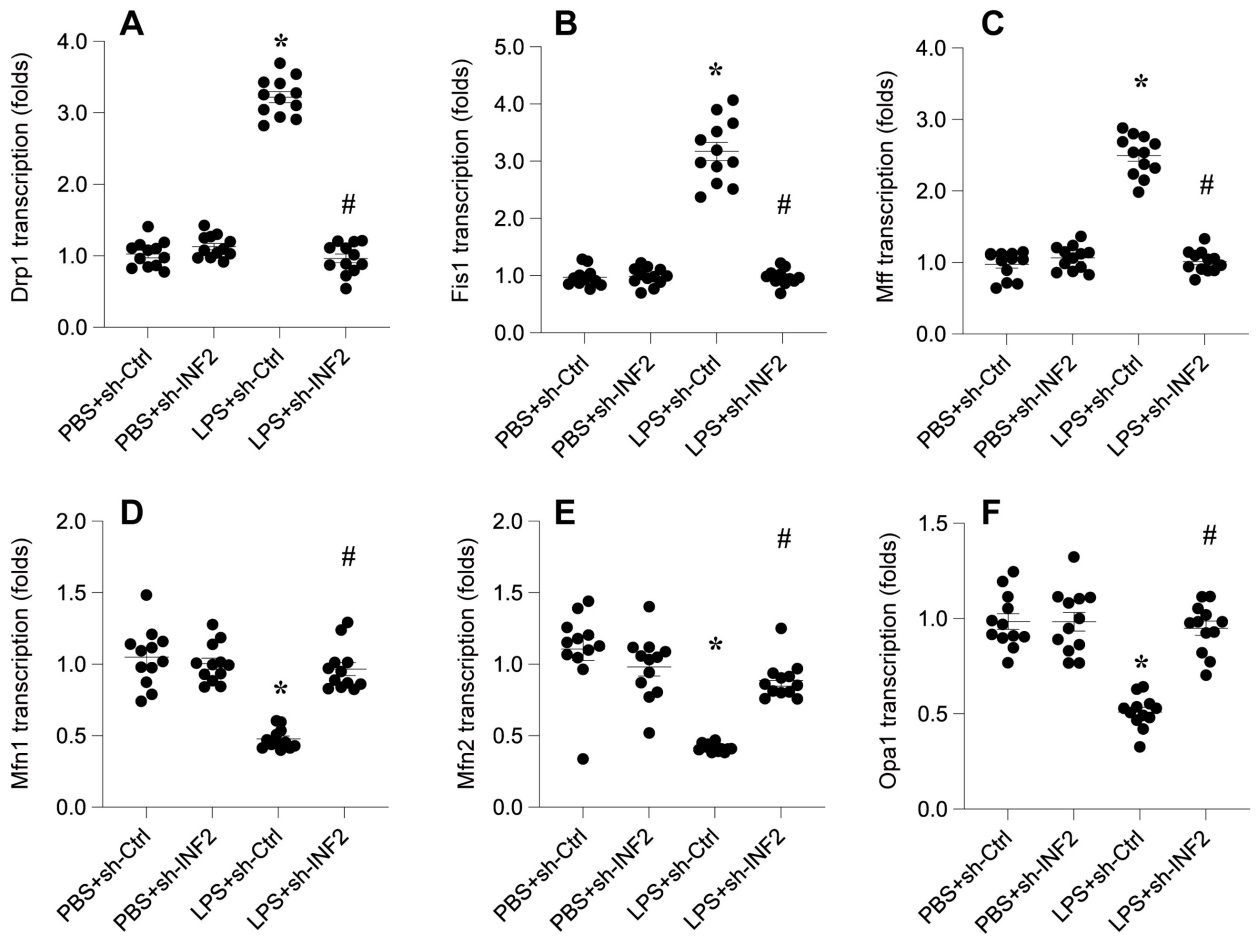
** INF2 knockdown attenuates LPS-caused mitochondrial dynamics disorder. A-F.** RNA was isolated from HL-1 cells and the transcriptions of Drp1, Fis1, Mff, Mfn1, Mfn2 and Opa1 were measured by qPCR. *p<0.05 vs. PBS+sh-Ctrl group, #p<0.05 vs. LPS+sh-Ctrl group.

Quantitative PCR (qPCR) analysis was conducted to assess the transcriptional changes of key mitochondrial dynamics genes following LPS exposure. The results showed a significant upregulation in the transcription of mitochondrial fission genes Drp1, Fis1, and Mff, post-LPS treatment (Figure 4A-F). Concurrently, there was a notable downregulation in the mRNA expression of mitochondrial fusion genes Mfn1, Mfn2, and Opa1, indicative of a shift towards mitochondrial fission in response to LPS (Figure 4A-F).

Remarkably, the knockdown of INF2 exhibited a protective effect against these LPS-induced alterations in mitochondrial dynamics. INF2 deletion not only prevented the LPS-mediated enhancement of mitochondrial fission but also facilitated the reversal of mitochondrial fusion processes (Figure 4A-F). This finding highlights the crucial role of INF2 in modulating the balance between mitochondrial fission and fusion, particularly in the context of LPS-related cellular stress.

These observations provide compelling evidence of INF2's regulatory influence on mitochondrial dynamics in septic conditions, offering new insights into the cellular mechanisms underpinning mitochondrial dysfunction during sepsis.

### INF2 knockdown attenuates LPS-caused mitochondrial dysfunction

Subsequently, we investigated the role of Inverted Formin 2 (INF2) in lipopolysaccharide (LPS)-mediated cardiomyocyte dysfunction, particularly focusing on its influence on mitochondrial homeostasis. The alterations in mitochondrial function were extensively analyzed in the context of LPS exposure and subsequent INF2 modulation.

**Figure 5:**
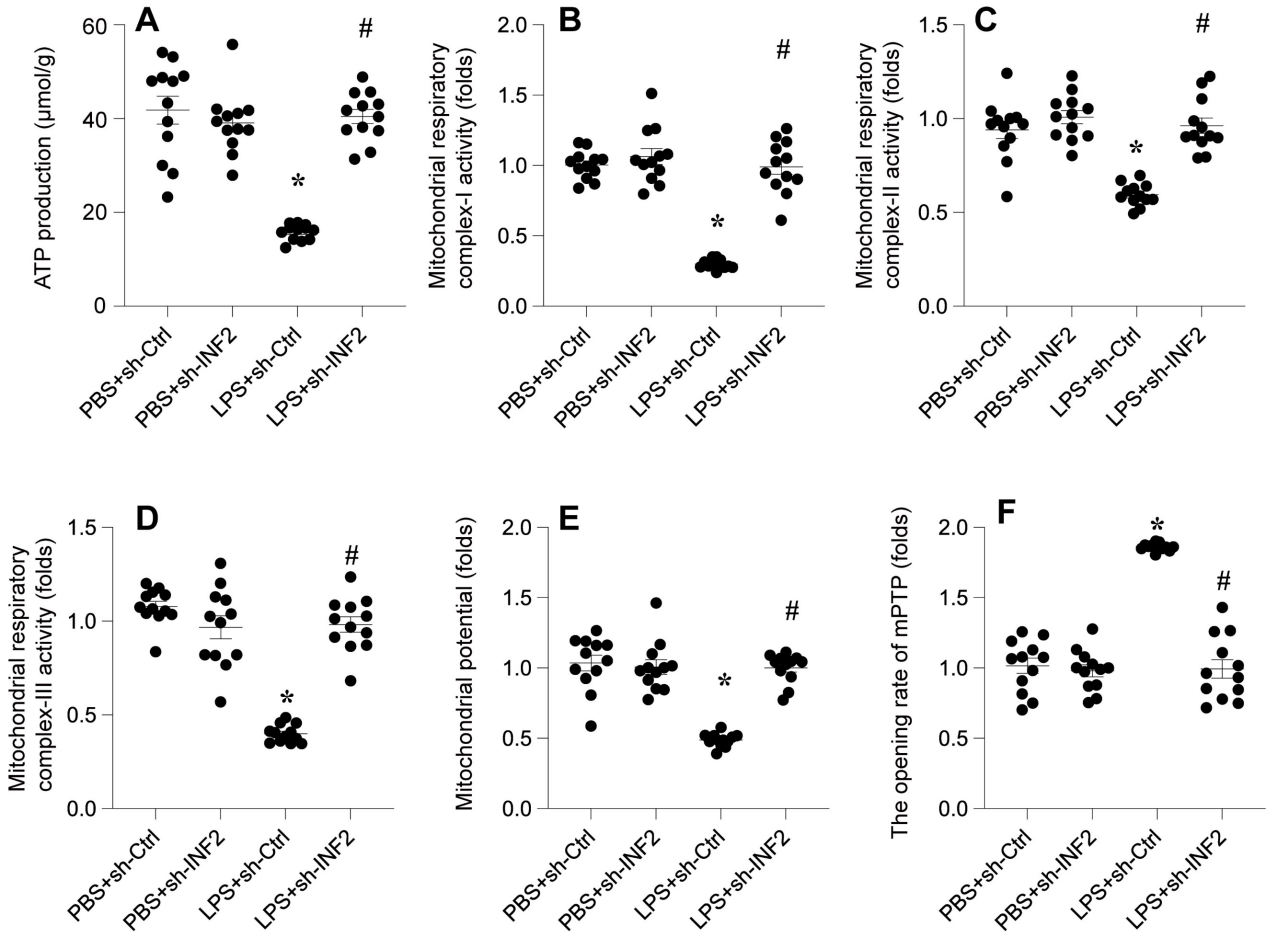
** INF2 knockdown attenuates LPS-caused mitochondrial dysfunction. A.** ATP production was determined by ELISA. **B-D.** The activities of mitochondrial respiratory complex I, II, III were measured by ELISA. **E.** Mitochondrial potential was evaluated through immunofluorescence assay and the relative changes of mitochondrial potential was evaluated. **F.** The mPTP opening rate was determined by an ELIST kit. *p<0.05 vs. PBS+sh-Ctrl group, #p<0.05 vs. LPS+sh-Ctrl group.

Initially, we observed a rapid decline in ATP production following LPS treatment, a hallmark of mitochondrial dysfunction (Figure 5A). Notably, this decline was significantly reversed upon the knockdown of INF2, indicating its vital role in maintaining mitochondrial ATP synthesis (Figure 5A). Given that ATP production is intricately linked to mitochondrial respiration, we utilized ELISA kits to evaluate the activities of key mitochondrial respiratory complexes I, II, and III. In response to LPS, a marked suppression of these complexes was evident (Figure 5B-D). However, this suppression was notably abrogated in HL-1 cells transfected with shRNA targeting INF2 (Figure 5B-D), suggesting a protective role of INF2 against LPS-induced mitochondrial respiratory impairment.

Furthermore, we assessed the mitochondrial membrane potential, another critical indicator of mitochondrial health. LPS exposure led to a reduction in membrane potential, which was not observed in cells with INF2 knockdown (Figure 5E). Additionally, we investigated mitochondrial apoptosis through the dynamics of mitochondrial permeability transition pore (mPTP) opening. LPS treatment significantly increased the mPTP opening rate (Figure 5F), a response that was attenuated by INF2 loss through targeted shRNA transfection.

Collectively, these findings illuminate the significant association between LPS-induced mitochondrial dysfunction and INF2 activation. This underscores the potential of INF2 as a crucial mediator in mitochondrial homeostasis and a promising therapeutic target in the context of septic cardiomyocyte dysfunction.

### INF2 knockdown reduces LPS-mediated cardiomyocyte dysfunction and death

**Figure 6:**
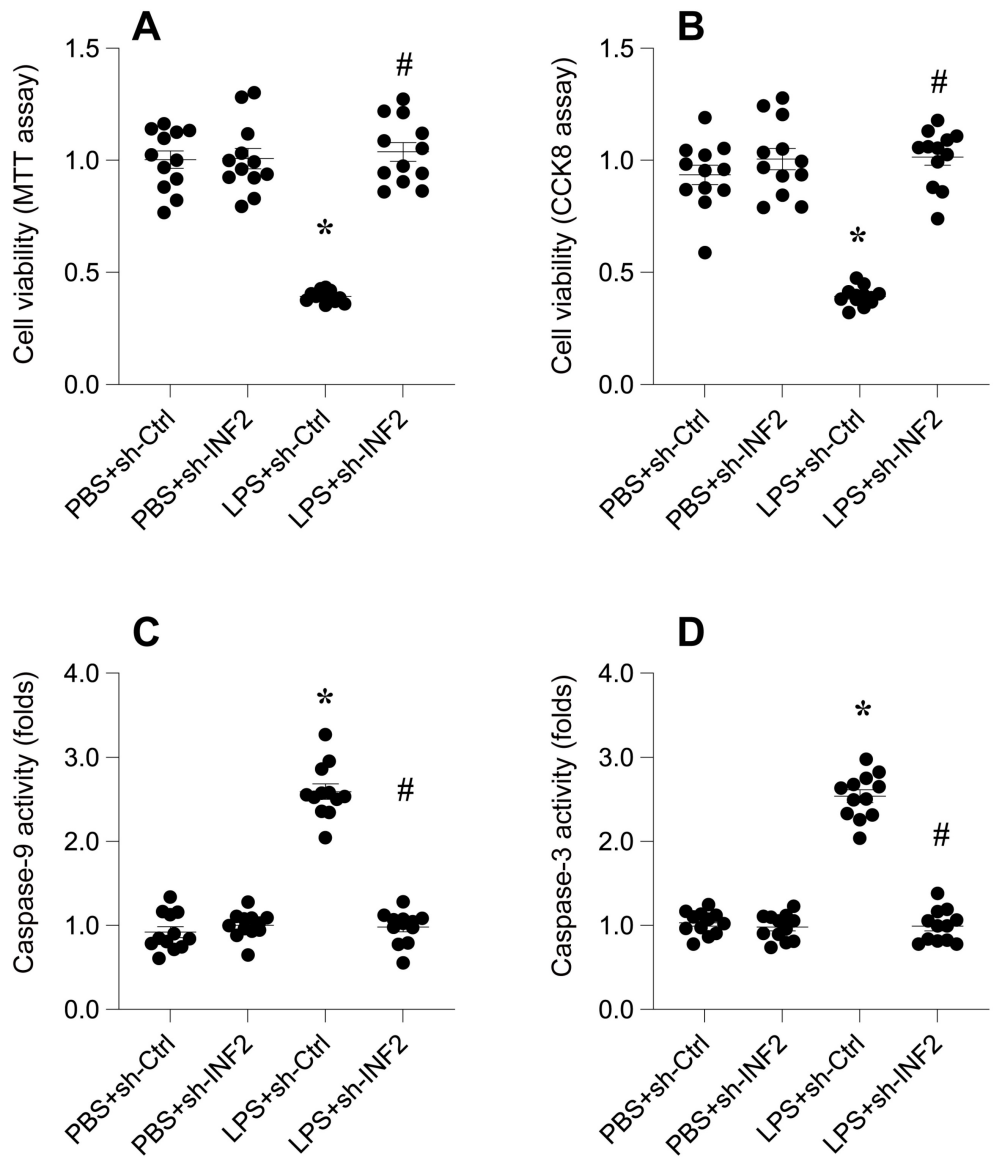
** INF2 knockdown reduces LPS-mediated cardiomyocyte dysfunction and death. A.** MTT assay was used to evaluate the cell viability of HL-1 cells in the presence of LPS. shRNA against INF2 was transfected into HL-1 cells before LPS treatment. **B.** CCK-8 assay was applied to analyze the cell viability of HL-1 cells upon LPS exposure. shRNA against INF2 was transfected into HL-1 cells before LPS treatment. **C-D.** ELISA kits were used to analyze the activities of caspase-3 and caspase-9 in the presence of LPS. *p<0.05 vs. PBS+sh-Ctrl group, #p<0.05 vs. LPS+sh-Ctrl group.

Next, we aimed to decipher the role of Inverted Formin 2 (INF2) in the pathogenesis of lipopolysaccharide (LPS)-induced septic cardiomyopathy. Utilizing HL-1 cardiomyocytes as an in vitro model, cells were exposed to 10 μg/ml LPS for 24 hours. Subsequently, cell viability was quantitatively assessed using both MTT and CCK-8 assays. As indicated in Figure 6A-B, LPS treatment resulted in a significant reduction in cell viability compared to the phosphate-buffered saline (PBS)-treated control group, suggesting a cytotoxic effect of LPS on cardiomyocytes.

To explore the involvement of INF2 in this LPS-induced reduction in cell viability, HL-1 cells were transfected with shRNA targeting INF2 prior to LPS exposure. Post-transfection, a reassessment of cell viability revealed a notable improvement in cells where INF2 was knocked down (Figure 6A-B), indicating a protective effect against LPS-induced cytotoxicity.

Further analysis was conducted using an ELISA kit to measure the activities of caspase-3 and caspase-9, key markers of apoptosis. Figure 6C-D show that LPS exposure significantly elevated the activities of these caspases. However, this activation was substantially mitigated upon INF2 knockdown, as evidenced by reduced caspase activities (Figure 6C-D).

These collective findings demonstrate that INF2 plays a critical role in mediating the deleterious effects of LPS on cardiomyocyte viability and function. The knockdown of INF2 not only improved cell survival but also impeded the activation of apoptotic pathways, underscoring its potential as a therapeutic target in septic cardiomyopathy.

## Discussion

The study focuses on the role of DNA-dependent protein kinase catalytic subunit (DNA-PKcs) in sepsis-induced cardiomyopathy (SIC). It highlights DNA-PKcs's contribution to cardiac dysfunction during sepsis by exacerbating inflammatory responses. The research reveals that reducing DNA-PKcs expression can alleviate cardiomyocyte death and dysfunction caused by lipopolysaccharide (LPS). Additionally, the study connects DNA-PKcs to mitochondrial dynamics by showing its regulatory effect on Inverted Formin 2 (INF2) expression. This novel insight into mitochondrial dysfunction in SIC suggests potential therapeutic targets in DNA-PKcs and INF2.

In the study, the role of DNA-PKcs in sepsis-induced cardiomyopathy (SIC) is examined, highlighting its influence on inflammation and mitochondrial dysfunction in cardiac cells. The research emphasizes the mitigation of lipopolysaccharide-induced cardiomyocyte damage through DNA-PKcs silencing, and its regulatory impact on Inverted Formin 2 (INF2), a factor crucial for mitochondrial dynamics. These findings are significant as they offer new insights into the pathophysiology of SIC and suggest potential therapeutic targets involving DNA-PKcs and INF2, promising avenues for treating mitochondrial-related cardiac dysfunctions.

DNA-PKcs, traditionally recognized for its role in DNA repair, plays a significant role in mitochondrial regulation and function. Emerging evidence suggests that DNA-PKcs modulates mitochondrial dynamics, impacting cellular metabolism and energy homeostasis 25. Its involvement in oxidative phosphorylation and reactive oxygen species (ROS) production links DNA-PKcs to mitochondrial health and stress responses 26. DNA-PKcs's influence extends to mitochondrial biogenesis and apoptosis, highlighting its potential as a therapeutic target in diseases where mitochondrial dysfunction is a key factor 27, 28. This underscores the complexity of DNA-PKcs's function beyond genomic maintenance, revealing its critical role in cellular energetics and mitochondrial integrity.

The study's findings about DNA-PKcs in sepsis-induced cardiomyopathy (SIC) should be integrated with existing literature by comparing and contrasting them with prior research on DNA-PKcs, mitochondrial dynamics, and cardiac dysfunction in sepsis. This involves discussing how the study's insights into the role of DNA-PKcs and Inverted Formin 2 (INF2) in mitochondrial dysfunction during SIC align with, or offer new perspectives to, the current understanding in the field. The integration should highlight the novelty and significance of the study's findings within the broader context of cardiac health research.

Inverted Formin 2 (INF2) has emerged as a crucial player in the realm of inflammatory diseases, a field where understanding cellular mechanisms is key to developing effective therapies. INF2, known for its role in actin cytoskeleton dynamics, also significantly influences mitochondrial function and stress response pathways 19, 29, 30. Recent studies suggest that INF2's dysregulation contributes to inflammatory processes by altering mitochondrial dynamics 31, 32, which are essential for cellular homeostasis and immune responses. INF2's involvement in modulating mitochondrial fission and fusion highlights its potential as a novel therapeutic target in inflammatory diseases. Its role in these diseases underscores a complex interplay between cytoskeletal dynamics, mitochondrial function, and cellular inflammation, opening new avenues for research and treatment strategies in the context of chronic inflammatory conditions.

Mitochondria play a pivotal role in the pathology of sepsis-induced cardiomyopathy, a condition where the heart muscle is weakened by systemic infection. In this context, mitochondria are not merely energy producers but also key players ins cellular signaling and stress responses 33. Their involvement extends from energy metabolism disruptions to the activation of cell death pathways 34. During sepsis, mitochondria face increased oxidative stress, leading to altered mitochondrial dynamics, including changes in fission and fusion processes. These changes contribute significantly to cardiac dysfunction, making mitochondria a potential therapeutic target. Understanding mitochondrial behavior in sepsis-induced cardiomyopathy could lead to novel treatment strategies, potentially improving outcomes in this challenging condition.

To discuss the clinical and therapeutic implications of the study's findings on DNA-PKcs and INF2 in sepsis-induced cardiomyopathy (SIC), focus on how these discoveries could influence future treatment strategies. Consider the potential of targeting DNA-PKcs and INF2 for therapeutic interventions, the implications for patient care in cases of SIC, and how these findings might pave the way for new approaches in mitigating mitochondrial dysfunction in cardiac diseases. This section should address the translational aspect of the research, bridging the gap between laboratory findings and clinical application.

## Funding

This study is supported by the NSFC (NO. 82000243).
